# Empirical and Rational Design of T Cell Receptor-Based Immunotherapies

**DOI:** 10.3389/fimmu.2020.585385

**Published:** 2021-01-25

**Authors:** Heather F. Jones, Zaki Molvi, Martin G. Klatt, Tao Dao, David A. Scheinberg

**Affiliations:** ^1^ Molecular Pharmacology Program, Memorial Sloan Kettering Cancer Center, New York, NY, United States; ^2^ Weill Cornell Medicine, New York, NY, United States; ^3^ Immunology Program, Memorial Sloan Kettering Cancer Center, New York, NY, United States

**Keywords:** T cell receptor, bispecific T cell engager, cross-reactivity, tumor infiltrating lymphocytes, immune mobilizing monoclonal T cell receptors against cancer, peptide- major histocompatibility complexes, T cell receptor mimic monoclonal antibody, T cell receptor-T cell

## Abstract

The use of T cells reactive with intracellular tumor-associated or tumor-specific antigens has been a promising strategy for cancer immunotherapies in the past three decades, but the approach has been constrained by a limited understanding of the T cell receptor’s (TCR) complex functions and specificities. Newer TCR and T cell-based approaches are in development, including engineered adoptive T cells with enhanced TCR affinities, TCR mimic antibodies, and T cell-redirecting bispecific agents. These new therapeutic modalities are exciting opportunities by which TCR recognition can be further exploited for therapeutic benefit. In this review we summarize the development of TCR-based therapeutic strategies and focus on balancing efficacy and potency versus specificity, and hence, possible toxicity, of these powerful therapeutic modalities.

## Introduction

Harnessing potent cellular effectors, such as cytotoxic T cells, and soluble molecules of the human immune system has become a successful strategy in the treatment of cancers of a variety of types. While often effective and generally well-tolerated, these effectors are not truly specific for the tumor. Typically, these therapies can either broadly activate cellular effectors, such as with interleukins, interferons, or checkpoint blockade antibodies, or are directed to lineage markers or cell surface differentiation antigens also found on normal cells and tissues. For example, monoclonal antibody (mAb) and chimeric antigen receptor (CAR) T cell therapies have emerged as some of the most successful and important strategies in cancer therapy. However, these modalities are traditionally reactive with a limited repertoire of extracellular antigens. For many cancers, appropriate antigens have not been identified. In contrast, the TCR evolved to detect subtle changes in cellular proteins that can include viral peptides or mutated oncogenic proteins. Thus, TCR-based agents can be directed to the vast majority of truly tumor-specific antigens, or relatively specific tumor-associated proteins, which are derived from intracellular proteins (1–3). Peptides derived from proteins of any subcellular location are presented on the cell surface in the context of major histocompatibility complexes (MHC), known as human leukocyte antigens (HLA) when referring to human MHC, where they are recognized by T cells through their TCRs (3). TCR-based therapies are able to recognize and react to cells expressing these mutated or differentially expressed, cancer-associated proteins presented on MHC. The exploitation of this powerful modality to treat cancer and possibly other serious diseases is dependent on understanding the unique features of their recognition and effector activities, the types of structures that can be developed to take advantage of these functions, and the possible liabilities that these molecules carry.

**Figure 1:**
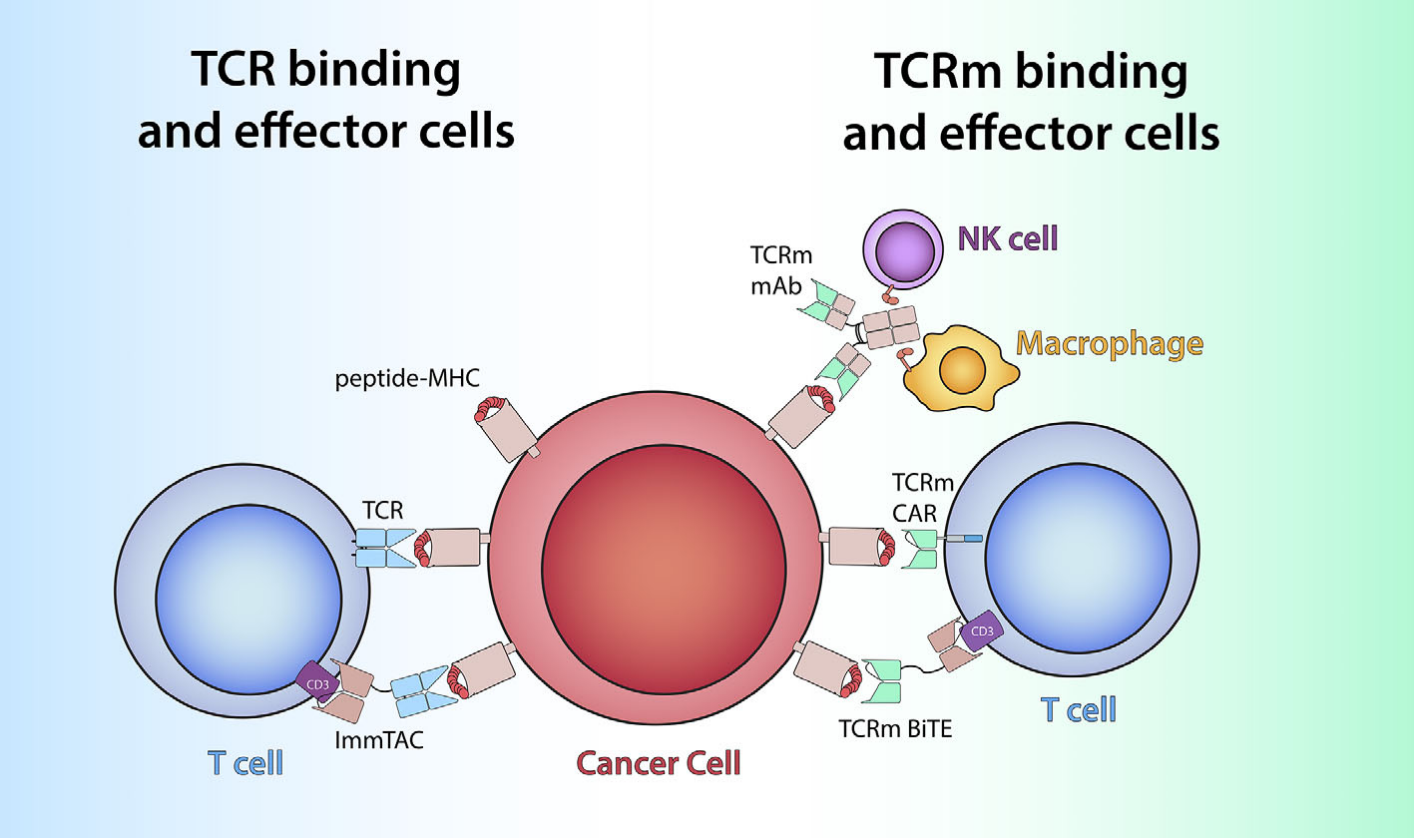
TCR-based therapeutics recognize peptide/MHC antigens (red and pink) on cells by utilizing either TCRs (light blue) or TCRm antigen-binding domains (green). Left: Soluble ImmTAC molecules bind peptide/MHC on cancer cells *via* alpha/beta TCR heterodimer similar to membrane-bound TCR and redirect the T cells by engaging extracellular CD3-epsilon (purple) *via* an anti-CD3 scFv. Right: TCRm mAb recognize peptide/MHC complex *via* its variable region (green) and to engage effector cells such as NK cells and macrophages to elicit Fc-receptor (orange) mediated ADCC or ADCP. TCRm CAR and bispecific mAb leverage TCRm-derived scFv to harness T cell effector function via engagement with intracellular CD3-zeta (blue) or extracellular CD3-epsilon (purple), respectively.

Immunotherapeutic modalities that take advantage of the TCR’s unique ability to recognize intracellular proteins are both molded by and constrained by key aspects of their structural features and those of their targets, as well as the origins of their antigenic specificity. Critical determinants of success for these agents are (1) the characteristics of the epitope (2); the affinity, avidity, and cellular geometry of the TCR; and (3) the recognition specificity unique to the antigen-TCR interaction. These features of TCRs are markedly divergent from the analogous features of antibodies and must be tackled accordingly to create a successful agent. First, unlike the conformational structure of the molecular targets of antibodies, the potential amino acid sequence epitopes for these TCR agents must be appropriately, expressed, processed, and presented on the cell surface. While peptide presentation on MHC molecules can be predicted in silico, these approaches are inaccurate and ideally, selected epitopes should be validated by using mass spectrometry to verify peptide-MHC presentation and followed by *in vitro* assays to characterize the functionality of target-specific T cells. Second, although unmodified, native TCRs reactive with peptides in context with their MHC proteins are more likely to yield appropriate specificity and functionality that mimic the actions of an endogenous T cell, as compared to a modified TCR, such native TCRs have orders of magnitude lower affinity than antibodies, which can limit their pharmacologic uses. TCRs may need affinity enhancement to increase the peptide-MHC recognition. In addition, native TCRs, unlike antibodies that operate in solution, cooperate as a collection of molecules along with other proteins in a cell membrane synapse on the T cell that vastly alters their effector functions. Third, TCRs, because of their low affinity and the complex structure of their epitope targets, are far more promiscuous than antibodies; strategies to predict toxicities by determining on-target/off-tumor and off-target antigen recognition of TCR-based agents are essential to ensure TCR agent safety, but such methods are currently in their infancy. There are no marketed drugs in the United States that are based upon the TCR. In this review, we will discuss various approaches to identify, address and overcome these constraints to TCR-based agents in order to advance these innovative drugs to clinical trials (**Table 1**; **Figure 1**).

**Table 1:** TCR-based agents in development.

TCR-Based Agent	Salient Features
Vaccines (many approaches)	Peptides and immunostimulants activate and expand antigen-specific preexisting T cells.
TILs and native T cells	T cells derived from patients or their tumors; patient-specific; often specific to an individual’s cancer.
TCR T cells	A specific TCR (native, foreign, or enhanced) transduced into a T cell.
ImmTac	A defined TCR single chain molecule linked to an scFv to CD3 to redirect T cells to cancers.
TCR mimic antibodies	Immunoglobulins (as IgG, bispecific antibodies, or incorporated into CAR T cells) directed to the peptide-MHC complex.

TIL, tumor infiltrating lymphocyte; ImmTAC, immune mobilizing monoclonal TCRs against cancer; scFV, single chain variable fragment; IgG, immunoglobulin G.

## Identification of Targets of TCR-Based Agents

Overall, advancements in screening techniques and engineering now provide multiple approaches and formats to achieve the goal of peptide-MHC recognition to target antigens. However, insufficient processing and presentation of the targeted epitope on the cell surface may limit activity. This underlines the importance of validation of target epitopes to ensure high levels of tumor specificity and efficacy. Ideally this can be achieved in advance by immunoprecipitation of MHC complexes and subsequent mass spectrometry identification of the displayed ligands.

The landscape of targets for TCR therapy of non-viral malignancies is comprised of antigens that demarcate aberrant cells, albeit to a highly variable degree (4). This nuance renders TCR target selection non-trivial. For the purposes of this review, we will divide TCR targets into two broad classes: self-antigens, derived from overexpressed proteins, and neoantigens, which exhibit subtle deviations from self due to the malignant state (5). The common feature between these two is that both are derived from the human genome; however, neoantigens arise directly from genomic deviations caused by the genomic instability of cancer.

The earliest class of cancer antigens known to be recognized by TCRs include self-antigens derived from proteins that are over-presented by MHC in tumor tissue. Classic examples that have been extensively studied are MART-1, Wilms’ tumor-1 (WT1), PR1, MAGE-A3, NY-ESO-1, carcinoembryonic antigen (CEA) and PRAME (6–8). However, an important distinction is that some of these are cancer-associated by means of their lineage-specificity, such as MART-1 and CEA, whereas others are cancer germline antigens, such as NY-ESO-1 and PRAME, that are only expressed in immune privileged sites such as the placenta or testis, but are re-expressed due to genomic instability in tumor cells (9). Lineage associated antigens require careful consideration of on-target/off-tumor effects associated with TCR therapy (7, 10). In contrast, TCRs targeting cancer germline antigens may confer greater tumor-specific recognition, but may be attenuated by escape mechanisms as these are not typically oncogenes critical for tumor survival (5). As this type of tumor-associated antigen has been studied for decades many of the used targets were also confirmed to be presented on the cell surface by MHC ligand isolation and mass spectrometry which renders them *bona fide* targets (11–13). The growing number of studies utilizing mass spectrometry to verify the presentation of HLA ligands is expected to increase confidence in presented epitopes derived from cancer germline antigens and potentially uncover new epitopes against which new TCR therapeutics can be developed.

More recently neoantigens, peptide antigens that are the result of missense mutations, frameshift mutations, or post-translational modifications, have been exploited as novel targets for TCR-based immunotherapies. However, other types of alterations can produce a neoantigen (14). Neoantigens encoded by missense mutations harbor a nonsynonymous amino acid substitution produced by either a driver or passenger mutation that can be distinguished by T cells by means of an augmented MHC binding affinity or altered TCR recognition. Frameshift mutations produce neoantigens that can be drastically different from wildtype protein-derived peptides, harboring multiple amino acid changes (15). Post-translational amino acid modifications, such as phosphorylation and glycosylation, can exhibit a greatly altered capacity to bind MHC and present a fundamentally different binding moiety to T cells compared to its unmodified variant (16, 17). Careful consideration of a tumor’s biologic properties is also necessary to guide effective target selection. For example, for hematologic malignancies self-antigens such as WT1, PR1, or PRAME, remain among the most useful TCR targets for these tumors rather than rare mutant neoantigens (18–22).

Neoantigens derived from frameshift mutations represent a type of shared neoantigen that is particularly prominent in clear cell renal cell carcinoma (ccRCC) in which neoantigens from common tumor suppressors such as TP53, PTEN, MLL2, MLL3 and ARID1A have been observed (23) and found to be recognized by tumor-infiltrating lymphocytes (TIL) (24). Strikingly, the immunogenic frameshift neoantigens were more distinct from the human proteome than were immunogenic missense mutations, a distinction that has also been made for immunogenic frameshifts in microsatellite instability-high tumors. Because of their high degree of sharing between tumors, immunogenicity, and derivation from driver mutations, frameshift-derived neoantigens may constitute a rational TCR target for tumors with adequate frameshift load.

Post-translationally modified peptides are an emerging class of neoantigens, the discovery of which has been accelerated by recent advances in mass spectrometry (25). Early reports discovered shared phosphorylated peptides (phosphopeptides) presented by tumor cell lines (26, 27) and leukemias (28). Phosphopeptides are an attractive target due to their consistently observed immunogenicity in normal donors, enhanced binding properties, and relationship to aberrant cancer metabolism (16, 28, 29). A recent phosphopeptide vaccine trial in melanoma patients elicited T cell reactivity in patients, suggesting that immune responses to these targets can be generated by tumor-bearing hosts (30). Glycosylated and acetylated peptides have similarly been shown to be immunogenic epitopes presented by tumor cells in similar studies (17, 31, 32). Proteomic data are expected to give depth to the cancer-specific modified peptide repertoire and provide a valuable link between post-translationally modified antigen presentation and tumor metabolism with additional classes of tumor-specific intracellular antigens identified and validated.

Although solid tumors present self-antigens, the efficacy of immune checkpoint blockade in highly mutated solid tumors such as melanoma and non-small cell lung carcinoma (NSCLC) has shifted interest to targeting one of the numerous neoantigens. Correlative studies have repeatedly demonstrated that a critical factor of response to checkpoint blockade is tumor mutational burden, with highly mutated tumors being more likely to respond (33, 34). This paradigm has been further validated by the discovery of T cells that recognize missense-derived neoantigens in responding patients (35). Subsequently, tumor exome sequencing and MHC binding predictions have become an invaluable tool to determine neoantigen load on a patient-by-patient basis (36). However, the accrual of neoantigens as a tumor evolves is unpredictable. Most neoantigens are derived from passenger mutations private to each individual patient or each tumor, rendering scalable TCR targeting infeasible. Furthermore, though some patient specific neoepitopes can be detected by mass spectrometry and their presentation therefore validated, the vast majority of predicted neoepitopes cannot be identified by this technique which further complicates target selection for patient-specific therapeutic approaches (37, 38). Neoantigens derived from shared driver mutations, such as KRAS and PIK3CA missense mutants where presentation has been validated for some HLA alleles (39), might overcome this issue (40–42).

## Regulation of Epitope Presentation

One potential disadvantage of targeting peptides presented by MHC molecules is their paucity of density on the cell surface, which may be 100 to 1000-fold lower than other antigens. This can be further exacerbated by down regulation of MHC by cancer cells as a method of immune escape (43). To deal with this issue, the immunopeptidome can be altered by drug or cytokine treatment to either augment expression or investigate the emergence of novel targets. Physiological alterations by interferon gamma (IFNγ) or tumor necrosis factor alpha (TNFα) favor the presentation of longer peptides as well as ligands which preferably bind to HLA-B alleles (44, 45). In addition to upregulating the HLA expression level, IFNγ also can lead to the specific increase in presentation of a given TCR or TCR-mimic monoclonal antibody (TCRm) peptide target through induction of the cytoplasmic immunoproteasome (46–49). This specific induction of ligand presentation could be utilized to render previously unreactive cells targetable (46).

Interestingly, ALK, RET, MEK, and other mitogen-activated protein kinase (MAPK) pathway inhibitors lead not only to a significant increase of HLA complex surface expression, which can help overcome immune escape *via* downregulation of HLA, but also to a large qualitative change of the displayed peptides (43, 50, 51). Many of which are potentially immunogenic epitopes. Surprisingly, ALK and RET inhibitor treatment can also lead to the presentation of T cell epitopes associated with impaired peptide processing (TEIPPs) which are usually only observed in transporter associated with antigen processing (TAP)-deficient or downregulated cells with low HLA levels. Treatment with the MEK inhibitor trametinib illustrates an example of improved efficacy of a specific TCR-like drug targeting an epitope from the MART-1 protein (50).

The ligandome might be affected to improve presentation of a specific TCR target by use of the proteasome inhibitor carfilzomib, which disfavors presentation of HLA ligands with aromatic C-terminal anchors by altering proteasomal cleavage patterns, as well as the ERAP1 inhibitor, DG013A, which augments presentation of ligands with higher affinity and shorter peptide length by changing endoplasmic peptide trimming (52, 53). Other chemotherapeutic agents, such as gemcitabine can lead to changes through upregulation of HLA-A,B,C complexes and also through immunoproteasome induction (54) or oxaliplatin, which can increase detection by CD4+ T cells through class II peptide presentation (55).

Hypomethylating drugs alone or in combination with histone deacetylase (HDAC) inhibitors are the most effective class of drugs that increase the presentation of specific HLA ligands. This has been most extensively demonstrated in acute myeloid leukemia (AML) for NY-ESO-1, MAGE-A3, and MAGE-A6 (56). Reinduction of cancer germline antigens through reversion of genetic repression marks is also feasible in many solid cancers, e.g. in esophageal squamous cell carcinoma for MAGE-A3 (57), ovarian cancer for protein expressed in prostate, ovary, testis and placenta (POTE) genes (58), mesothelioma for NY-ESO-1, MAGE-A1, MAGE-A3, and XAGE-1b (59) and prostate cancer for NY-ESO-1 (60). Since the presentation of HLA ligands can be altered qualitatively and quantitatively through multiple FDA-approved drugs, therapeutic strategies may benefit from combination therapies of TCR-based agents with HDAC inhibitors and hypomethylating drugs. The synergistic effects of HDAC inhibitors and hypomethylating drugs have been shown to be especially important for immunotherapies as the repressive marks on relevant genes can often only be sufficiently reversed by such a combination treatment (61).

## Generation of Therapeutic T Cells

T cells reactive with tumor associated antigens and neoantigens have been found in TILs and peripheral blood lymphocytes (PBL) (62, 63) of cancer patients, as well as in PBLs of healthy donors (64). Such cells, or their TCRs, would be expected to be an appropriate source for effective therapeutic agents. Despite this, many endogenous T cells, including those found within tumors, are still unable to eradicate tumors presenting their cognate antigen. This failure can be attributed in part to the immunosuppressive tumor microenvironment (TME) (3), low affinity of endogenous T cell receptors (TCR) for tumor associated antigens (65, 66), and possibly other factors. Numerous approaches have been employed to generate a more potent anti-tumor T cell response. Tumor-reactive T cells expressing native TCRs can be stimulated *in vivo* through administration of vaccines, checkpoint blockade inhibitors, or cytokines. Alternatively, reactive T cells can be expanded ex vivo and reinfused for adoptive cell therapy (67). Tumor-reactive T cells can be enriched and used in bulk for treatment, or their individual reactive TCRs can be sequenced and subsequently expressed exogenously in T cells prior to reinfusion.

### Cancer Vaccines

Naturally occurring tumor-reactive T cells can be stimulated to boost the anti-tumor T cell response through vaccination with tumor antigens. Vaccines can be peptides (68), DNA or RNA products (69), whole proteins, viruses encoding antigenic peptides (68) or autologous dendritic cells presenting peptide antigens (68, 70). Patients may receive a personalized cancer vaccine, where target peptides are chosen from tumor-specific mutations identified by whole exome sequencing (WES) and filtered through HLA binding prediction (69–71). Some peptides used for vaccination can be modified to further increase their immunogenicity, by substituting peptide residues to result in better binding to HLA molecules. Such “heteroclitic” peptides have been shown to induce stronger T cell responses that cross-react with their native sequences. Characteristic examples are the HLA-A*02:01 restricted peptides, NY-ESO-1 (SLLMWITQC) and WT1 (RMFPNAPYL), where replacing the final amino acid with valine (72), or the first amino acid with tyrosine (73), respectively, increases the HLA binding affinity resulting in increased immunogenicity and enhanced T cell activation. Cancer vaccines have been shown to increase the preexisting anti-tumor T cell response and have proven effective in some patients (68–70, 74–76). Although cancer vaccines are a widely used approach in investigational clinical trials, due to their limited clinical efficacy to date, more potent, passive therapeutic approaches using TCR recognition of tumor antigens have been developed in recent years. This review will focus on these new approaches.

### Adoptive T Cell Therapy

Tumor reactive T cells, present either in TILs or PBLs, can be removed from patients for rapid expansion ex vivo, outside of the immunosuppressive TME (77), prior to use for adoptive cell therapy (67). T cells can be stimulated for expansion with resected tumor (67, 78, 79), antibodies targeting CD3 and CD28 (80), or peptide antigens (81, 82). Synthetic peptides of neoepitopes identified by whole exome or RNA sequencing and subsequent HLA binding prediction can be pulsed onto (64, 83–85) or expressed as tandem minigenes (84, 86, 87) on antigen presenting cells, often autologous dendritic cells. For tandem minigenes, mutations are flanked by sequences encoding endogenous amino acids allowing the peptides to be processed and presented on MHC. When using tandem minigenes the reactive peptide and MHC restriction of the respective neoepitope does not have to be identified and it allows multiple antigens to be expressed and presented on the same cell (77, 84, 86, 87). Peptide stimulation has also been employed to generate reactive T cells from the peripheral blood of HLA matched healthy donors (64, 77, 88).

T cells with predefined antigen specificity can be isolated using peptide-MHC multimers (62, 63, 77, 89–94) through either magnetic enrichment or fluorescence activated cell sorting (FACS) when both the antigen and the MHC it binds are known or predicted. Patient specific neoantigens can be predicted based on WES data (94, 95). Multimers have been used to identify antigen specific T cells from patient TILs and PBLs (63, 95) and from healthy donor PBLs (91, 94). In contrast, cell surface biomarkers on T cells can be used to identify reactive T cells without knowledge of the specific antigen or the HLA on which it is presented (96–100). Several biomarkers, including PD-1 (96, 101, 102), LAG-3, TIM-3 (102), OX40 (103), CD137 (84, 97–100, 102–104) and CD107a (105) and cytokine production, such as of IFNγ, indicate the T cell has interacted with its cognate antigen and can be used to isolate T cells (106). Such markers have been used to identify tumor reactive cells from both TILs and PBLs (96). While these approaches have successfully identified tumor reactive T cells, there are limitations. Not all T cells that are multimer positive, and therefore peptide-MHC specific, are able to exert cytotoxic effects against tumor cells expressing these antigens (105). Conversely, multimer staining may not detect all antigen-reactive T cells (107–109). This can be due to decreased TCR surface density or expression of TCRs with low affinity. This can be especially problematic as TCRs reactive with self-antigens and MHC class II antigens tend to have lower affinity for their target. Multimer staining can be enhanced to detect low-affinity TCRs with protein kinase inhibitors, such as dasatinib which decreases TCR downregulation, cross linking antibodies to stabilize multimer binding, anti-coreceptor antibodies, and staining with multimers with more peptide-MHC sites, i.e. dextramers or dodecamers over tetramers (107, 109). Additionally, cytokine production and cytotoxicity are independently regulated, therefor cytokine production does not always correlate with cytotoxic potential (105, 110). These limitations can make it more challenging to accurately identify tumor-specific cytotoxic T cells.

### TCR Gene Therapy

Isolated individual reactive TCRs can be transduced and expressed into other T cells, known as TCR-T cells, to broaden therapy to additional patients. Paired TCR alpha and beta chain sequences can be identified from tumor-reactive T cells for subsequent cloning into expression vectors from pooled T cell (111, 112) or single cell (77, 83, 89, 92, 97, 113) sequencing data. Other methods to generate reactive TCRs for subsequent identification circumvent thymic selection to generate high affinity T cells reactive against specific tumor antigens. Immunization of mice expressing human HLA molecules (42, 114) or mice expressing the human TCR repertoire can be used for immunization and isolation of high affinity TCRs (115, 116). High affinity human TCRs with increased activity also can be isolated when human T cells are stimulated ex vivo with tumor antigens on HLA mismatched antigen presenting cells (117–120).

Alternatively, individual TCRs can be affinity enhanced *via* protein engineering to increase their anti-tumor effects (7, 65, 121, 122). As few as one or two amino acid changes in the complementarity determining regions can increase the affinity of TCRs (8, 65, 114, 123), evident by slower TCR off rates (124). High throughput methods such as phage (124, 125), yeast (126, 127), and T cell display libraries (128, 129), along with somatic hypermutation (130), and in-vitro T cell differentiation (131) have been employed to generate high affinity TCRs, sometimes in conjunction with available structure data (132). While increasing TCR affinity has been shown to increase the effectiveness of the T cell (65, 74, 123), TCRs whose affinities are too high can become less effective (115) and are at higher risk for cross reactivity (74, 115, 123). Drawing any direct correlation between TCR affinity and T cell efficacy can be challenging as affinity itself is determined based on two parameters, the on- and off-rate of TCR binding (133). One model describes T cell responses as requiring long enough dwell time between the TCR and peptide-MHC to stimulate signaling but having a fast enough off-rate to allow for sequential TCR binding and signaling amplification (134, 135). Therefore longer TCR/peptide-MHC half-lives may prevent serial triggering and hamper T cell responses (134). Additionally, mechanisms that decrease the efficacy of T cells have been identified in association with T cells with high affinity TCRs (136). These mechanisms include impaired T cell signaling, upregulation of the inhibitory receptors, such as PD-1, down regulation of costimulatory receptors (135), peripheral deletion, expansion of anergic T cells (136, 137) and TCR down regulation (136).

Finally, T cell therapies can be designed to target patient-specific tumor antigens or public tumor antigens. T cell responses against patient-specific mutated neoantigens have been associated with clinical successes (83, 87, 138) and should be subject to less central tolerance, as such neoantigens are not present in normal tissues (102, 139). Neoantigen-reactive T cells can be highly tumor specific as T cells are able to distinguish between single amino acid changes in peptides, representing either unmutated self or mutant peptide sequences (95). While targeting neoantigens is expected to result in less toxicities (97, 102, 140), finding tumor and patient-specific antigens and reactive TCRs to generate patient specific TCR-T cells is challenging, costly and not currently feasible on a broad scale. Public tumor antigens are not patient or cancer-specific and while they sometimes can be derived from mutant peptides (140, 141), they are often unmutated self-peptides from tumor associated proteins that are minimally or not expressed on normal cells (142, 143). Public antigens have the benefit that the same TCR construct can be used to treat multiple patients (74).

## Specificity of TCR-Based Therapies

One the most important questions for clinical application of TCR-based agents is specificity, in order to prevent off-target toxicities. T cells and other TCR-based therapies rely on precise recognition of a short linear peptide sequence, typically 8 to 11 amino acids in length in the groove of a largely structurally constrained HLA class I protein (144). Therefore, the TCR must be able to distinguish between the different antigenic peptides derived from thousands of proteins, which may comprise highly similar amino acid sequences, challenging absolute specificity. The estimated 100 million different TCRs expressed by a human is dwarfed by the number of potential sequence targets in the proteome. Therefore, it is speculated that each TCR can recognize hundreds to thousands of different antigens (145). In this way, TCR promiscuity can be a source of both greater scope of protection, but also significant off-target toxicity. Additionally, native TCRs can have low, micromolar affinity for their cognate target, especially if they are targeting non-mutated peptides, due to thymic selection (65, 66). While increasing affinity, as described above, may increase the anti-tumor effects of the TCR, bypassing thymic selection increases the risk for off-target reactivity and toxicity (122) and hence, a balance between TCR activity and toxicity must be struck. The severe off-target toxicities sometimes seen with TCR therapies has emphasized the need for methods to predict reactive off-target peptides and their cells of origin (65, 142).

### On-Target/Off-Tumor Toxicity

TCR-based therapies can lead to autoimmune toxicities caused by on-target/off-tumor responses, which occurs when the target antigen is expressed on normal cells. On-target/off-tumor autoimmune toxicity has been seen in some melanoma patients treated with exogenously expanded TILs, which recognized non-malignant melanocytes, as with T cells reactive against melanocyte differentiation antigens, such as MART-1, and with DMF5 TCR-T cells, specifically reactive with the MART-1 antigen (10, 113, 146). Patients variably experienced uveitis, rash, vitiligo, and hearing loss (147). Interestingly these toxicities are seen with DMF5 TCR T cells, but not with T cells expressing the lower affinity DMF4 TCR (143, 147), reactive with the same MART-1 epitope. The higher affinity TCR, DMF5, is hence both more efficacious and more toxic (147). On-target/off-tumor colitis has additionally been seen with T cells using a TCR developed in an HLA-A*02:01 humanized mouse, affinity enhanced for binding to a CEA epitope presented on HLA-A*02:01, likely due to CEA expression on gastrointestinal cells (7).

### Molecular Mimicry and Sequence Similarity Toxicity

Molecular mimicry is when a peptide is able to stimulate TCR reactivity due to structural similarities with the target peptide. An example of molecular mimicry was observed with the affinity enhanced a3a TCR, reactive with a MAGE-A3 peptide on HLA-A*01 (65). While pre-clinical screening showed no evidence of cross reactivity, after TCR-T cell therapy patients died from cardiac failure, which was later attributed to cross reactivity with a peptide from the cardiac protein titin (65). The MAGE-A3 peptide target, EVDPIGHLY, and the Titin peptide target, ESDPIVAQY, differ in four amino acid positions, some of which are in the center of the peptide, the area principally responsible for contact with the TCR (66). Existing methods were unable to predict the cross reactivity preclinically (65, 66). Hence, better methods to predict off-target reactivity for TCRs is an unmet need.

An affinity enhanced ImmTAC designed from the same parent TCR as the a3a TCR was also found to have cross reactivity with the titin peptide. Tissue cross reactivity was still observed with a TCR agent that was more specific to the MAGE-A3 epitope and was attributed to high levels of titin expression on myoblasts (66). Therefore, protein expression is another variable that needs to be considered when testing TCR-based therapies for cross reactivity, as well as affinity and half-life of the TCR/peptide-MHC interaction (66). Additional instances of cross reactivity have been seen due to sequence similarity, as seen with a TCR against a HLA-A*02:01 restricted MAGE-A3 peptide which recognized a peptide unpredicted to be expressed in the brain and led to neurotoxicity including death (114, 142).

### Mixed TCR Dimers

If the endogenous TCR alpha or beta chain pairs with an exogenously introduced alpha or beta chain, the resulting TCR, a mixed TCR, could have unknown reactivity with normal peptides (148). These mixed TCRs bypass thymic selection, therefore there is no central tolerance to prevent reactivity with normal tissues (148). To prevent mixed TCR dimer formation, the human constant regions can be interchanged with murine constant regions (122, 147, 149–151), or human TCR alpha and beta constant regions can be interchanged with each other or with the constant regions from gamma-delta T cells, which cannot pair with endogenous alpha-beta chains (152). Additionally, constant regions can be modified to contain cysteines to promote disulfide bond formation and therefor pairing between the alpha and beta chains (85, 148). Other methods to prevent TCR chain mispairing involve transduction of alpha-beta TCRs into gamma-delta T cells (153, 154) and knocking out or down the endogenous TCR chains with clustered regularly interspaced short palindromic repeats (CRISPR)/CRISPR associated protein 9 (Cas9) (155) or small interfering RNA respectively (156–158). TCR mispairing can lead to off-target as well as decreased on-target activity (85, 149). Promoting proper TCR chain pairing increases TCR expression (85, 122, 149), avidity and activity (122).

## Predicting Off-Targets of TCR-Based Therapeutic Agents

**Figure 2:**
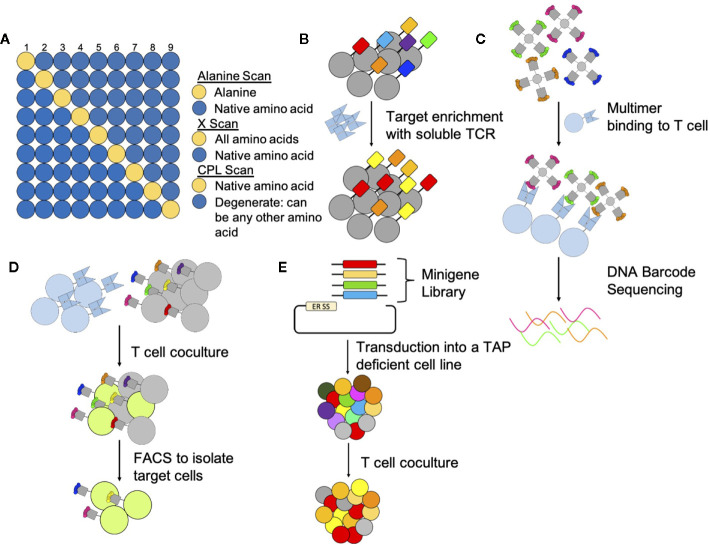
Methods to determine targets and off targets of TCR-based therapeutics. **(A)** Peptide Scans: Peptide scanning techniques determine which residues are important and in which positions. Alanine scans and x-scans hold all positions in the native peptide (blue) except one (yellow); one position is switched to an alanine for alanine scans or to all other amino acids for X-scans. CPL scans hold one position constant (yellow) and all other positions (blue) can be any combination of amino acids. Peptides are then pulsed onto target cells to assay for TCR reactivity. **(B)** Yeast Display: Yeast display libraries genetically encode a peptide linked to an MHC. Multiple rounds of selection with soluble TCRs select for peptide-MHC complexes that are recognized by the TCR. **(C)** Multimers: Multimers can identify T cells that bind specific peptide-MHC complexes. To identify reactive peptide-MHC complexes in a pooled setting multimers are DNA barcoded prior to incubation with and binding to T cells. **(D)** T scan and SABR: The T Scan method genetically encodes for longer peptides that go through endogenous processing and presentation. Target cells that present peptide-MHC complexes targeted by T cells fluoresce through a granzyme reporter system and are subsequently sorted by FACS. For the SABR method, TCR expressing cells bind to target cells expressing the SABR receptor. After TCR binding, this receptor, which consists of an MHC linked to a CD3z and CD28 domain, signals to an NFAT report system causing target cells to fluoresce. Target cells can then be sorted by FACS. **(E)** PresentER: The PresentER system genetically encodes for peptides to be presented in MHC complexes on TAP-deficient cells. Target peptides are identified through coculture depletion assays with T cells.

Prediction of TCR reactivities has proven difficult, as native TCRs are necessarily cross-reactive to enable tissue surveillance. Additionally, TCR reactivity is structurally complex being dependent on the quality and quantity of the expression of the TCR, MHC and presented peptides (1). Steps to detect cross reactive TCRs include screening for reactivity against cell lines known to express the HLA of interest, but not the proteins from which the target peptide is derived (65, 159), as well as HLA mismatched cell lines (159, 160). Reactivity with either of these cells would indicate off-target binding of the TCR and a potential for toxicity. A number of investigations have sought to predict cross-reactivity *via* structural analyses and predictive algorithms. Single amino acid replacement scans, such as alanine scans (**Figure 2A**), are often used to identify peptide residues important for TCR recognition (159, 161–163). Alanine scans involve changing every position in a peptide sequence to an alanine, if reactivity is abolished then that particular amino acid residue and its position are considered necessary for TCR reactivity. However, alanine screens do not identify important interactions if the substituted amino acid is similar to alanine. Changing that amino acid to alanine might still result in binding and therefore alanine screens are biased towards identification of TCR interactions with large and charged residues where the substitution to alanine resembles a major change. A second single amino acid replacement scan can be performed to provide a more complete picture of motif residues important for TCR reactivity (65, 160). This motif can subsequently be compared to human peptides (161), to identify putative cross-reactivities (65).

Other amino acid scans screen additional amino acid combinations to determine the TCR binding motif. X-scans (**Figure 2A**), similar to alanine scans, hold all peptide positions constant except one but change this position to any of the remaining 19 amino acids (162). Combinatorial peptide libraries (CPL) (**Figure 2A**) are another peptide screening method to determine TCR off-targets which allow additional peptide diversity. CPLs are designed so that one position of a peptide is held constant and the remaining positions are changed to any other amino acid (145, 161, 164). Peptides from CPL scans are screened in subpools (145) for TCR reactivity (161, 165). X-scans and CPLs allow for more potentially cross-reactive peptides to be screened compared to alanine scans providing a more complete picture and ranking of potential TCR reactive peptides (161, 162, 165, 166). In the aforementioned amino acid scans, T cell reactivity can be measured in numerous ways including *via* cytokine release (145, 161, 162), typically measuring IFNγ, T cell proliferation (164), target cell lysis (164, 166), detection of T cells activation markers and binding of soluble TCRs to peptide-MHC complexes (166).

Computational modeling methods take previously known TCR and peptide-MHC structural and reactivity data to create models to predict peptide-MHC targets (167–169) and cross reactivities (170). These models are based on the premise that TCRs with common targets will have structural and sequence similarities (167, 171). Characteristics used to compare TCRs include length, charge, hydrophobicity and sequence (171). Peptide-MHC complexes have also been compared to assess cross reactivity based on charge and available surface area (170). These methods are primarily limited by the amount of data available for model development and TCR comparison (167, 168).

However, these structural or predictive methods discussed above are neither comprehensive nor fully accurate, as the rules for binding of TCRs to their peptide/MHC sites are still poorly understood. Therefore, more empiric methods have been applied to the problem. Large libraries, where the peptide target is genetically encoded into expression systems have been used to identify TCR targets and off-targets. Display libraries of peptides have been developed for screening in yeast and baculovirus systems (**Figure 2B**) (172, 173). In these systems the MHC is expressed with the peptide attached by a linker (144, 172–174). For these systems the MHC must fold properly, and the peptide must bind the MHC properly (172). In another library system, known as PresentER (**Figure 2E**), peptides are directed to be loaded onto the endogenous MHC of mammalian cells through an endoplasmic reticulum signaling sequence. Target peptides are identified after coculture screens with T cells by assaying for peptide dropout *via* DNA sequencing (175). This system has the advantages of yielding actual peptide-MHC molecules in the context of human cell surface membranes on live cells for both *in vitro* and *in vivo* work, as well as allowing functional assays such as recognition and killing of targets to be measured, but is limited in the number of peptides that can be scanned in a single assay to a few tens of thousands, whereas the proteome may contain a million potential epitope sequences that bind to an individual MHC. An additional library screening technique uses signaling and antigen-presenting bifunctional receptors (SABR) (**Figure 2D**), where the target cell expresses peptides linked to MHC receptors fused to intracellular CD3ζ and CD28 domains. The target cells are identified through fluorescence, as these cells have an NFAT-GFP reporter system which is activated upon signaling from CD3ζ after TCR engagement. The presented target peptides are subsequently identified through sequencing. As with PresentER libraries, SABR libraries are limited in their size. However, SABR libraries can contain up to one million epitopes (176). The previously described libraries genetically encode for short antigenic peptides, the T-scan reporter system (**Figure 2D**) on the other hand encodes for larger amino acid sequences that need to undergo endogenous processing and presentation (177). Trogocytosis, which describes the facilitated exchange of membrane bound proteins after immune cells and target cells come into close contact, has also been used to identify TCR targets in library screens. This mechanism then allows for the identification of the recognized antigen presented on target cells, that were engaged by a T cell (178). Other library screening methods use DNA-barcoded MHC multimers (**Figure 2C**). The MHC multimer is screened for binding with a TCR followed by sequencing of the DNA barcode to determine which peptides were able to bind the TCR of interest or to develop recognition motifs to predict additional off targets (163). Such methods may prove useful in the preclinical characterization of TCR reactivity and could be paired with tissue expression data of off-target genes to predict site-specific toxicities.

## Current TCR-Based Cellular Agents in Clinical Study

The earliest studies in which TCR-T cells were infused into patients were reported in 2006 (143), an effort that was the culmination of decades of work by Rosenberg and colleagues to characterize the antitumor activity of TILs (179–181). In this early study, TCRs specific for the HLA-A*02:01 presented self-antigens MART-1, gp100, NY-ESO-1, and p53 were transduced into autologous peripheral blood mononuclear cells (PBMC) and infused into melanoma patients. TCRs specific for these antigens are among the most actively studied in TCR gene therapy clinical trials.

Notably, NY-ESO-1-reactive TCRs are being investigated by multiple academic and industry entities worldwide for the treatment of a range of solid and liquid tumors such as melanoma, sarcomas, lung cancers, and multiple myeloma (8, 121, 182, 183). GSK3377794, comprised of autologous T cells transduced with an affinity-matured NY-ESO-1–reactive TCR (184) has reached Phase Ib/II trials testing it in combination with checkpoint inhibitor, anti-PD1, therapy in NSCLC (NCT03709706).

WT1-directed TCRs have also shown promise for treatment of AML (185). WT1 has been designated a highly prioritized antigen (186), and the cytotoxicity of WT1-specific T cells against leukemic cells has been reported by multiple groups (6, 20, 187). WT1-specific T cells can be readily generated from most healthy donors; accordingly, a TCR isolated from a healthy donor could be used without enhancement of its native antigen-binding capability. Moreover, donor-derived Epstein-Barr virus-specific T cells were transduced with the TCR, rather than autologous T cells. This study is notable in several aspects as it utilizes a healthy donor-derived TCR, genetic modification of allogeneic T cells, and provides validation of the preclinical work involved in characterizing WT1 as a leukemia-associated antigen (188, 189). Results from two WT1 TCR Phase I/II trials utilizing autologous T cells are expected to clarify the relationship, if any, between graft versus host disease (GvHD), which has been seen in trials, and WT1-targeting (NCT02550535, NCT01621724, NCT02408016).

A class II-restricted TCR directed against an epitope of MAGE-A3 presented by HLA-DP*04:01/04:02 was shown to be well-tolerated and the CD4 autologous T cells persisted in 17 patients with metastatic cancer in a basket trial (190). Three partial responses in a variety of cancers correlated with T cell persistence of at least one month. Though this TCR was derived from a regulatory T cell clone (191), infused T cells did not appear to differentiate to regulatory T cells (Treg) on the basis of FOXP3 expression. The safety profile of this TCR contrasts that of previous MAGE-A3 TCRs (142, 192), and thus could prove to be an effective therapy with minimal toxicity in a wide range of tumors.

Of particular concern in TCR gene therapy is the safety of affinity enhanced self-antigen TCRs due to potential on and off-target toxicities (10, 123). The TCR targeting an HLA-A*02:01 presented epitope of CEA, described earlier, was found to induce severe colitis in all three colorectal cancer patients tested (7, 193) because of baseline CEA expression in colonic mucosa. An affinity enhanced TCR specific for MAGE-A3 (114) was found to cause severe neurotoxicity due to reactivity with a similar MAGE-A12 epitope (142). Another affinity enhanced TCR to MAGE-A3 caused lethal cardiotoxicity due to recognition of a titin-derived epitope (65, 192). These cases exemplify the critical need for characterizing TCR target recognition before clinical translation.

The safety of neoantigen reactive TCRs appears to be in stark contrast to that of the aforementioned self-antigen reactive TCRs because neoantigens derived from private somatic mutations are theoretically only presented by the tumor. In addition to tumor-selectivity, the potential for acquired resistance to TCR therapies targeting such mutations are expected to be lower in the case of targeting driver mutations. For example, T cells targeting mutant KRAS found in the endogenous TILs of a patient with KRAS-driven metastatic colorectal cancer showed minimal toxicity and all seven of the patient’s lesions initially regressed. Interestingly, at 9 months a lesion escaped by means of downregulating the restricting HLA allele, while maintaining the same KRAS mutation (141). Hence, KRAS driver mutations are not easily mutated into escape variants, but downregulation of HLA can be an alternative mechanism of immune escape. To this end, KRAS mutant-specific TCRs have been generated in HLA transgenic mice and are currently being tested in phase I trials (42) (NCT03190941). Similarly, T cell responses to TP53 hotspot mutations have been found in TILs in different epithelial cancers with multiple HLA allele restrictions (84, 194). The recurrence of TP53 mutations encoding immunogenic neoantigens presents profound opportunities for TCR-based therapy across a variety of solid tumors (195).

Several TCR-transduced cells are being tested in ongoing or recently completed Phase I and II trials (**Table 2**). New antigens being targeted in these trials include HERV-E and TRAIL-DR4. New TCR modalities are being assessed as well, such as suicide gene-containing T cells that provide a kill-switch and Vγ9Vδ2 TCR-transduced T cells which recognize uncharacterized tumor antigens in an MHC-independent manner.

**Table 2:** Selected TCRs and T cells in ongoing or recently completed clinical trials.

Trial ID	Target	HLA allele	Phase	Industry partner	Citations	Notes and Indication(s)
NCT01586403	Tyrosinase	A*02:01	I		(196)	Melanoma
NCT03399448	NY-ESO-1	A*02:01	I	Tmunity Therapeutics	(197)	CRISPR-edited to replace endogeneous TCR Myeloma, melanoma, sarcoma
NCT01343043	NY-ESO-1	A*02:01 A*02:05 A*02:06	I	GlaxoSmithKline	(198)	Affinity enhanced TCR Synovial Sarcoma
NTR6541	Unknown; CD277-mediated presentation	N/A	I	Gadeta	(199)	Vγ9Vδ2 TCR AML, MDS, MM
NCT03132922	MAGE-A4	A*02	I	Adaptimmune Therapeutics	(200)	Affinity enhanced TCR Various solid tumors
NCT04044768	MAGE-A4	A*02	II	Adaptimmune Therapeutics	(200)	Affinity enhanced TCR Synovial Sarcoma or Myxoid/Round Cell Liposarcoma
UMIN000002395	MAGE-A4	A*24:02	I	Takara Bio	(201, 202)	A24 transgenic mouse-derived TCR Esophageal cancer
UMIN000011519	WT1	A*24:02	I	Takara Bio	(6, 203)	AML, MDS
NCT02592577; NCT02989064	MAGE-A10	A*02:01 A*02:06	I	Adaptimmune Therapeutics	(204)	Affinity enhanced TCR NSCLC, melanoma, head & neck
NCT03503968	PRAME	A*02:01	I/II	Medigene		AML, MDS, MM
NCT02743611	PRAME	A*02:01	I/II	Bellicum Pharmaceuticals	(205)	incorporates inducible caspase-9 suicide gene AML, MDS, melanoma
NCT03686124	PRAME	Undisclosed	I	Immatics		Solid tumors
NCT03925896	EBV LMP2	A*02 A*11 A*24	I			Nasopharyngeal carcinoma
NCT03971747	AFP	A*02:01	I	Cellular Biomedicine Group		HCC
NCT03441100	MAGE-A1	Undisclosed	I/II	Immatics		NSCLC, HCC
NCT03326921	HA-1	A*02:01	I			incorporates inducible caspase-9 suicide gene Acute leukemias
NCT03354390	HERV-E	A*11:01	I		(206)	Renal cell carcinoma
NCT02988258	CMV pp65	A*02:01	I		(207)	post-HSCT CMV disease
NCT02719782, NCT02686372	HBV	Various	I	Lion TCR	(208, 209)	HCC
NCT00923390	TRAIL-DR4	N/A	I		(210)	MHC-independent TCR Renal cell carcinoma
NCT00991224	HIV SL9	A*02	I	Adaptimmune Therapeutics		Affinity enhanced TCR HIV/AIDS
NCT03132792	AFP	A*02	I	Adaptimmune Therapeutics		HCC

NY-ESO-1, New York esophageal squamous cell carcinoma 1; HCC, hepatocellular carcinoma; MDS, myelodysplastic syndrome; MM, multiple myeloma; MAGE, melanoma associated antigen; PRAME, preferentially expressed antigen in melanoma; EBV, Epstein–Barr virus; LMP2, latent membrane protein 2; AFP, alpha fetoprotein; HERV-E, human endogenous retrovirus group E; CMV, cytomegalovirus; HSCT, hematopoietic stem cell transplantation; HBV, hepatitis B virus; TRAIL, tumor necrosis factor-related apoptosis-inducing ligand; DR, death receptor; HIV, human immunodeficiency virus; AIDS, acquired immunodeficiency syndrome.

## TCR-Based, Non-Cellular Therapies

Current T cell therapies using either TILs or TCR transduced T cells are patient-specific and require TCR gene transduction or expansion of the patient’s T cells *in vitro* before reinfusion into patients. Such processes have proven to be difficult to translate into a widely available therapy. Several therapeutic modalities (**Table 3**) have been developed to overcome such limitations and broaden therapeutic options to a wider range of patients. In particular, soluble T cell redirecting biologics based on either TCR or immunoglobulin molecules, in conjunction with redirection of powerful T cell cytotoxicity provides a promising alternative.

**Table 3:** Characteristics of TCR, TCRm and traditional mAb.

Feature of Agent	TCR	TCR mimic mAb	mAb
**Structure**	Heterodimer which functions in a complex and forms a synapse upon activation.	Various soluble Ig formats or as transmembrane CAR in cells.	Various soluble Ig formats or as transmembrane CAR in cells.
**Affinity (Typical)**	Micromolar or modified to nanomolar.	Picomolar to nanomolar	Picomolar to nanomolar
**Plasma kinetics**	Soluble forms are unstable alone. ImmTac half-life is 6–8 h	Half-life may be from hours to weeks based on structure.	Half-life may be from hours to weeks based on structure.
**Epitope targets**	Peptide-MHC complex on cell surface. Peptides derived from total proteome or may be viral or microbial in origin.	Peptide-MHC complex on cell surface. Peptides derived from total proteome or may be viral or microbial in origin.	Protein or carbohydrate on cell surface. Soluble proteins or other molecules. Limited to extracellular and secreted proteome. May also be viral or microbial components.
**Therapeutic formats available**	Bispecific forms or transduced as receptor into cell.	Native or modified IgG; BiTE and Bispecific forms; CAR; ADC; radioconjugate.	Native or modified IgG; BiTE and Bispecific forms; CAR; ADC; radioconjugate.
**Effector functions**	Directs T cells to kill.	Redirects T cells to kill; ADCC; ADCP; CDC; recruits NK cells or macrophages to kill. Can serve as a vehicle for drug or isotope delivery.	Neutralizes or activates signaling. Redirects T cells to kill; ADCC; ADCP; CDC; recruits NK cells or macrophages to kill. Can serve as a vehicle for drug or isotope delivery.
**Marketed agents**	None.	None.	Numerous, for multiple diseases.
**Minimal epitope number required**	May be a dozen or less	Tens to hundreds	Hundreds to Thousands and much higher

BiTE, bi-specific T cell-engager; ADC, antibody-drug conjugate; ADCC, antibody-dependent cellular cytotoxicity; ADCP, antibody-dependent cellular phagocytosis; CDC, complement-dependent cytotoxicity; NK, natural killer.

### Immune Mobilizing Monoclonal TCRs Against Cancer (ImmTac)

A new class of bi-specific molecules, ImmTacs, are soluble T cell engagers (sTE), designed to use a TCR specific for a peptide-HLA complex, genetically linked to a single chain variable fragment (scFv) of an anti-CD3 mAb. Structurally, an ImmTac begins with a human TCR or an affinity enhanced TCR. While recombinant soluble TCRs (lacking transmembrane and intracellular parts) should theoretically be an ideal therapeutic vehicle for targeting intracellular tumor antigens, TCRs are inherently unstable in soluble form and tend to form aggregates, which poses a significant technical challenge in developing such molecules as therapeutics (211). To address this issue, ImmTacs are designed to stabilize soluble TCRs through the incorporation of an interchain disulfide bond buried within the core of the TCR. Finally, an anti-CD3 scFv is encoded *via* a flexible linker to the beta chain of the TCR (212). Once TCRs engage their antigenic peptide-MHC complexes, the anti-CD3 effector arm mediates potent redirection of polyclonal T cells to the target. With this technology, cells expressing fewer than 100 copies of the targeted peptide-MHC complexes can be killed. Since natural TCRs have low, micromolar affinity, ImmTac technology allows increases in TCR affinity up to the subnanomolar range, allowing ImmTacs to target low density peptide-MHC complexes of intracytoplasmic tumor antigens (213). The most extensively studied ImmTac molecule, tebentafusp (IMCgp100), an affinity enhanced TCR specific for a gp100 peptide (a melanocyte differentiation antigen) presented on HLA-A*02 complexes, has demonstrated clinical efficacy as a monotherapy against the gp100-positive uveal melanoma (214, 215). In addition, three other molecules: GSK01 (directed to the cancer germline antigen NY-ESO-1), IMC-C103C (directed to cancer germline antigen MAGE-A4) and IMC-F106C (directed to a cancer testis antigen PRAME) are in phase I/II trials for treatment of multiple myeloma, melanoma and a range of other cancers (216). ImmTacs’ high potency and drug-like soluble format make them easy agents to develop and distribute widely.

### TCR-Mimic Monoclonal Antibody (TCRm)

MAb-based therapy has become one of the most successful and important strategies for the treatment of cancer and rheumatologic diseases (217, 218). MAbs are characterized by high target specificity, limited side effects and prolonged half-life *in vivo*. The intrinsic multifunctional cellular engagement of mAbs include antibody-dependent cellular cytotoxicity (ADCC), complement-dependent cytotoxicity (CDC), antibody-dependent cellular phagocytosis (ADCP), blocking of ligand-based signaling, and direct signaling or inhibition *via* various pathways. In addition to their intrinsic properties, mAbs can in an antigen-specific manner deliver potent cytotoxic agents such as toxins, drugs, or radionuclides to cancer cells (219). Finally, mAbs can be re-engineered to generate CARs or bi-specific antibodies to redirect the T cells or other effector cells for potent anticancer therapy (220). Commercial therapeutic mAbs are directed to extracellular or cell surface proteins; therefore, the vast majority of intracellular tumor-associated antigens (TAAs) are not addressed by FDA approved mAb therapy (221).

TCRms targeting peptide-MHC complexes, combine TCR recognition of peptide-MHC complexes, with the potency and versatility of mAb drugs. The well-characterized platform of TCRms provides a highly feasible method to target intracellular tumor antigens for a broad range of patients. Increasing advances in library screening technology allow rapid identification and selection of highly specific TCRms to intracellular tumor antigens. A number of mouse and human TCRm antibody fragments such as antigen binding fragments (Fab) or scFvs have been identified (222, 223), as well as several full-length human TCRms, and have been investigated as potential therapeutic agents. A murine hybridoma-generated TCRm (8F4) reactive with the myeloid leukemia antigen PR1-derived epitope (VLQELNVTV) was found to bind HLA-A*02:01 and was later humanized. This TCRm eliminated human AML in xenografts (224) and has now advanced to clinical trials. While active alone, to improve its potency 8F4 was engineered into a bi-specific T cell-engager (BiTE) to redirect polyclonal T cells to PR1-positive leukemias (225). The first fully human TCRm, ESK1, specific for a WT1-derived epitope/HLA-A*02:01 complex, was developed by our group (226). WT1 is expressed in a wide range of human cancers and has been an important target for TCR-based adoptive cell transfer of engineered T cells, as well as peptide, DNA, and dendritic cell vaccines (185, 227–231). WT1 expression encompasses both hematopoietic and solid tumors and therefore, a TCRm targeting this oncoprotein should have a broad application as a therapeutic agent against a variety of human leukemias, myeloma and solid tumors (226, 232).

Our group has described TCRms specific for a cancer germline antigen, PRAME, which is widely expressed in various cancers (recognizing ALYVDSLFFL/HLA-A*02:01) (46), and FOXP3, the hallmark protein for Tregs (recognizing TLIRWAILEA/HLA-A*02:01) (233). The TCRm specific for the FOXP3 epitope is particularly interesting because of the profoundly suppressive role of Tregs in the TME. Strategies of depleting Tregs by mAbs against surface proteins such as CD25, CCR4 and GITR, have not been successful thus far because these molecules are shared between Tregs and other immune effector cells (234–236). Other human TCRms to important cancer-associated and viral targets have been described (**Table 4**).

**Table 4:** Human TCRm reported.

Antigen target	HLA restriction	Indications and uses	Citations
Proteinase 3	A*02:01	Myeloid Leukemias; formatted as IgG and CAR T cell	(224, 225)
WT1	A*02:01	Leukemias and various solid tumors; formatted as IgG, BiTE, and CAR T cell	(226)
PRAME	A*02:01	Leukemias and various solid tumors; formatted as IgG, BiTE, and CAR T cell	(46)
FOXP3	A*02:01	Tregs, FOXP3+ T cell malignancies and other types of cancers; formatted as IgG, BiTE	(233)
Ras G12V	A*02:01	Wide range of solid tumors: pancreatic, colon, ovarian and more; formatted as IgG	(237)
Epstein Barr Virus	A*02:01	B cell lymphoma and carcinoma; formatted as IgG	(238)
WT1	A*24:02	Leukemias and various solid tumors; formatted as CAR T cell	(239)
Minor HA-H1	A*02:01	Leukemias; formatted as CAR T cell	(240)
AFP	A*02:01	Hepatic carcinoma; formatted as CAR T cell	(241)
hCG-beta	A*02:01	Ovarian, colon, and breast cancer; formatted as hIgG1, mIgG2a	(242)
NY-ESO-1	A*02:01	Melanoma and solid tumors; formatted as Fab, CAR T cell	(243, 244)
MAGE-A1	A*01:01	Melanoma; formatted as CAR T cell	(240)
GP100	A*02:01	Melanoma; formatted as CAR T cell	(240)
MUC-1	A*02:01	Breast cancer; formatted as Fab	(245)
hTERT	A*02:01	Melanoma and prostate cancer; formatted as Fab	(246)

FOXP3, forkhead box P3; hCG, human chorionic gonadotropin; GP100, glycoprotein 100; MUC-1, mucin 1; hTERT, human telomerase reverse transcriptase.

TCRm mAbs for proteinase 3 and AFP are currently in clinical trials.

The antibody-based format for TCRm offers the opportunity for optimization *via* protein engineering strategies to address different needs:

- Fragment crystallizable (Fc) region modification. The ADCC activity of mAbs can be enhanced 5-to 10-fold by Fc region protein engineering (247) or by modification of Fc-region glycosylation (248, 249).
- Bispecific mAbs (BsAb). BsAbs are designed to recognize two different epitopes or antigens, and they comprise a large family of molecules, with a wide variety of formats (250). Such bispecific molecules function by recruiting and activating polyclonal T cells or other effector cells. BiTEs are a subtype of BsAb, composed of a scFv specific for tumor antigen on one arm, linked to a scFv for CD3 on the other arm. Such a BiTE molecule functions by recruiting and activating polyclonal T cells at tumor sites, thereby bypassing MHC restriction and co-stimulation, while retaining epitope specificity needed for traditional TCRs (251). The ESK1-BiTE was the first TCRm-based BiTE, which showed superior cytotoxicity than an immunoglobulin form against a wide range of tumor cells expressing WT1 *in vitro* and *in vivo* in mice. The ESK1-BiTE also induced robust secondary CD8 T cell responses against other epitopes *via* epitope spreading (232). Such a mechanism may be important for long-lasting anti-tumor immunity by controlling the outgrowth of tumor cells that have lost the target protein or that have downregulated the primary target during tumor evolution. In addition, as a small molecule, BiTEs may penetrate more easily than CAR T cells into the TME of solid tumors, where it can bridge tumor targets with TILs.
- TCRm CAR T cells. CAR T cell constructs use immunoglobulin scFvs, recognizing extracellular cell surface protein antigens expressed by cancer cells. The scFv is linked to a transmembrane and intracellular signaling domain generally containing the CD3ζ chain of the TCR complex, as well as costimulatory domains of CD28 or 41BB. Following the clinical success of CD19 CAR T cell therapy in human leukemia (252), many CAR T cells have been developed targeting a variety of cell surface molecules (253). Using TCRms, described above, CAR T cells have been generated against the intracellular tumor antigen WT1, by use of the ESK1 TCRm scFv, thereby opening the door for CAR T cells to enter an entirely new universe of antigens (254). These studies show CAR T cells can be used to target intracellular tumor antigens, in contrast to CAR T cells using traditional scFVs that target extracellular proteins, and thus expand the utility of this platform to include a large majority of cancers. The first CAR T cells expressing a TCRm scFv for alpha fetoprotein (AFP)-HLA-A*02 recently advanced to human trials for the treatment of hepatocellular carcinoma. Currently, more CAR T cells are being developed from TCRms recognizing various epitopes from NY-ESO-1, gp100, and MAGE-A1, in the context of HLA molecules (240).
- Affinity maturation. Cell surface protein targets of mAbs normally have a high density of typically 10,000 to 1,000,000 molecules per cell. In contrast, intracellular tumor antigens presented as peptide-HLA complexes on the cell surface typically have low densities, often far less than a few hundred molecules (46, 226, 255, 256). Therefore, WT1-specific TCR gene therapy (185) and ImmTac molecules use affinity enhanced TCRs (212). Similarly, *in vitro* affinity maturation of mAbs, often with phage library technology, has successfully been used to optimize specific mAbs with increased affinity for their targets (257). Most TCRms have been derived using phage display technology and have yielded relatively high affinity TCRms, thereby reducing the need for affinity maturation.

### Challenges and Opportunities for Soluble TCR Constructs and TCRm

Soluble TCR-based agents represent novel classes of biologics that make immunotherapy accessible for some of the most interesting and highly tumor specific intracellular antigens and offer pharmacological and manufacturing advantages (**Table 3**). However, fundamental questions remain to be studied in order to further advance these drugs. First, given the intrinsic nature of TCR recognition of a linear peptide bound to HLAs, cross reactivity to other similar complexes is an important issue in all TCR-based therapies. Second, unlike TCR, TCRm are not natural structures that evolved with thymic selection to recognize peptide-HLA complexes. As antibodies are generally selected on membrane bound soluble proteins or carbohydrate antigens during B cell development *in vivo*, selection methods using phage or other libraries may introduce unnatural biases and unstable structures in addition to cross-reactivity. TCRm may never completely mimic natural TCR recognition. For example, crystallography studies have shown that the ESK1 Fab primarily interacts with N-terminal residue of the peptide and HLA-A*02:01 (258). An alanine substitution study showed that the TCRm mAb specific for the PRAME peptide-HLA-A*02:01 mainly recognized the C-terminal residues of the peptide (46). In general, TCRs dock onto peptide-HLA complexes using a conserved canonical binding mode, forming a large binding interface between the TCR and peptide-HLA, enabling broader contacts across both peptide backbones and HLA heavy chain. Lessons learned from the early development of TCRm could help optimize screening strategies of phage libraries to select ideal phage clones that more closely mimic TCR recognition. These strategies include selection of mAbs that bind with optimal valency between HLA and peptide, or mAbs that bind to a broader range of amino acid residues in the center of the peptide in a fashion similar to TCRs (259). Additionally, native TCRs tend to have orders of magnitude lower affinities than TCRm or engineered TCRs. The impact this has on activity and specificity, and the importance of affinity in different formats is not well understood. For example, a TCR-T cell or TCRm CAR T cell may need less affinity than a soluble format such as a BiTE due to its multivalent avidity. However, an anti-Ep-CAM BiTE has been shown to form a synapse after engaging its target because of the proximate contacts between effector and target cells (260). A recent study directly compared a scFv specific for NY-ESO-1p157/HLA-A*02:01 complex in BiTE and CAR-T cell formats. The conclusion was that the BiTE and CAR T cells showed a similar functional avidity, assessed by cytokine production and killing activity (261). A study comparing a TCR specific for a WT1-derived peptide/HLA-A*02:01 complex and a TCRm specific for the same complex showed that while the native low-affinity alpha-beta TCR maintained potent cytotoxic activity and specificity, the high-affinity TCR-like antibody CAR T cells exhibited reduced activity and loss of specificity. This TCR-like mAb in a monovalent or bivalent context maintained high specificity, however, when the avidity of this mAb was increased through expression in a CAR T cell format, it exhibited loss of specificity (262). This study suggested that TCRm is less suitable for CAR T cell format than being used as mAb format coupling with more potent drugs. However, function of each mAb depends on the specificity of the particular TCRm used for construction of the CAR T cells. Although this study raised an interesting question, one pair of TCRm vs TCR may not generalize the function of these two formats. Interestingly, a number of TCRms have been converted to a CAR T cell format (**Table 4**). With a rapidly growing numbers of these new modalities, detailed studies are required to address these fundamental biological questions.

In addition, despite all the advantages that TCR-based, non-cellular therapies offer, they also have certain limitations. Both ImmTacs and BiTEs have a short half-life (4 to 8 h), which requires continued administration of the agents (216, 232). However, this can be overcome by a growing number of engineering technologies.

## Summary and Perspective

TCR-based therapies provide a number of unique advantages over other immunotherapies, but also present challenges associated with their structures and the methods used for their generation. On and off-target identifications and toxicity prediction remain problematic. TCR-based agents are a powerful modality on which to create immunotherapies as the TCR is able to target the vast repertoire of cancer associated and mutated proteins found in all subcellular locations (3). The TCR’s unique and valuable recognition properties have been taken advantage of in adoptive cell therapies, where reactive T cells are enriched or T cells are modified to express reactive TCRs, and in non-cellular therapies, which bypass the extensive process of T cell enrichment, modification and expansion, while mimicking the peptide recognition properties of the TCR in the form of soluble TCRs or antibodies. In distinct contrast to traditional antibodies and CAR T cells, TCR-based therapies recognize a short peptide bound to an MHC found on the surface of cells (144). The TCR’s unique ability to recognize intracellular proteins in these complexes both shapes and constrains their functions. Moreover, the origins of their antigenic specificity are dependent on a linear sequence of amino acids that may be shared by other proteins in the proteome. In addition, the complicated and highly regulated process of antigen presentation means that not all peptides are presented, and others are displayed are at insufficient densities to trigger TCR-based recognition or responses. This problem may be overcome in some cases with small molecule drugs or cytokines and such a strategy could be considered for combination therapies. Endogenous TCRs are able to discern between peptides with single amino acid changes (95), enabling precise differentiation between peptides; despite this, TCRs are inherently promiscuous to enable proper immune surveillance, resulting in potential liabilities for their use therapeutically. Compounding this issue is that in silico predictions of peptide presentation on MHC molecules are inaccurate. Ideally, selected epitopes should be validated by using mass spectrometry to verify peptide-MHC presentation, yet these methods are costly and tedious.

While manipulating TCR affinity has been shown to increase their effectiveness (65, 74, 123) and would provide agents with better pharmacologic properties, TCRs whose affinities are too high risk cross reactivities (74, 115, 123). Additionally, affinity enhanced, modified and artificial TCR-based therapies do not necessarily adhere to the same binding rules as native TCRs, due to the absence of thymic selection. Therefore, toxicities from TCR-based therapies are of concern and methods to identify potential cross-reactive or off-target toxicities are imperative. Current preclinical strategies to predict toxicities by determining on-target/off-tumor and off-target antigens are largely empiric and are unable to cover the vast potential repertoire of epitopes in the proteome. These failures have led to clinical toxicity in early trials. To avoid toxicity, the targeting of neoantigens may be used, but identifying patient specific tumor antigens and reactive TCRs is challenging, costly and currently not feasible broadly. Alternatively, public tumor antigens offer broader applications, but are rare and may not be absolutely cancer specific.

Despite challenges to development, TCR-based therapies have shown great potential in clinical use, targeting seemly un-targetable intracellular proteins. In distinction to commercial therapeutic mAbs or CAR T cells, which are generally limited to a small number of extracellular or cell surface proteins; TCR-based agents allow, for the first time, access to the vast majority of intracellular tumor associated antigens (TAAs) that are not currently addressed by FDA approved therapies. It is expected that technologies to automate the identification of target and off-target epitopes, and rapid new methods to generate TCRs, as well as new soluble and cell bound structures that take advantage of the unique recognition properties of the TCR, will soon result in a great expansion in these agents to a broader population of patients with cancer and other diseases.

## Author Contributions

All authors contributed to the content and revisions of this review and approved the submitted version.

## Funding

This work was supported by the National Cancer Institute (F31 CA253995, P01 CA23766, R01 CA55349, R35 CA241894, and P30 CA08748), the Greenberg Fund, the Solomon Fund, the Tudor Fund, the Center for Experimental Therapeutics of MSKCC, and an Individual Research Grant from the German Research Foundation (KL 3118/1-1).

## Conflict of Interest

MSKCC has filed for patent protection on behalf of DAS and TD for inventions related to this paper. TD is an advisory board member for Eureka Therapeutics. DAS is an advisor for, or has equity in, Eureka Therapeutics, Oncopep, Sellas, Pfizer, and Iovance,

The remaining authors declare that the research was conducted in the absence of any commercial or financial relationships that could be construed as a potential conflict of interest.
